# Zebrafish Larvae Exhibit Rheotaxis and Can Escape a Continuous Suction Source Using Their Lateral Line

**DOI:** 10.1371/journal.pone.0036661

**Published:** 2012-05-03

**Authors:** Julia Olszewski, Melanie Haehnel, Masashige Taguchi, James C. Liao

**Affiliations:** 1 Department of Biology, The Whitney Laboratory for Marine Bioscience, University of Florida, Saint Augustine, Florida, United States of America; 2 Department of Ecology and Evolutionary Biology, Brown University, Providence, Rhode Island, United States of America; Center for Genomic Regulation, Spain

## Abstract

Zebrafish larvae show a robust behavior called rheotaxis, whereby they use their lateral line system to orient upstream in the presence of a steady current. At 5 days post fertilization, rheotactic larvae can detect and initiate a swimming burst away from a continuous point-source of suction. Burst distance and velocity increase when fish initiate bursts closer to the suction source where flow velocity is higher. We suggest that either the magnitude of the burst reflects the initial flow stimulus, or fish may continually sense flow during the burst to determine where to stop. By removing specific neuromasts of the posterior lateral line along the body, we show how the location and number of flow sensors play a role in detecting a continuous suction source. We show that the burst response critically depends on the presence of neuromasts on the tail. Flow information relayed by neuromasts appears to be involved in the selection of appropriate behavioral responses. We hypothesize that caudally located neuromasts may be preferentially connected to fast swimming spinal motor networks while rostrally located neuromasts are connected to slow swimming motor networks at an early age.

## Introduction

The mechanosensory lateral line system allows fishes to detect water flow relative to the body. The basis of this sensory system is an array of receptor units called neuromasts, which contain clusters of hair cells along with support cells. Upon deflection, hair cells increase transmitter release and excite afferent neurons [1]. While adult fishes may possess tens of thousands of neuromasts, larval zebrafish (*Danio rerio*) contain only about 24 on the surface of the body [2] at 5 days post fertilization (dpf). Even at this early developmental stage, zebrafish larvae exhibit several distinct motor behaviors which are mediated by the lateral line, such as turns, struggling and escape behaviors [3]–[8]. Due to its accessibility and tractability, the larval lateral line is uniquely suited for experimental studies involving selective neuromast ablations. This provides an attractive opportunity to manipulate neuromast number and arrangement in order to investigate the functional consequence of the spatial organization of flow sensors in an intact, freely-swimming animal.

**Figure 1:**
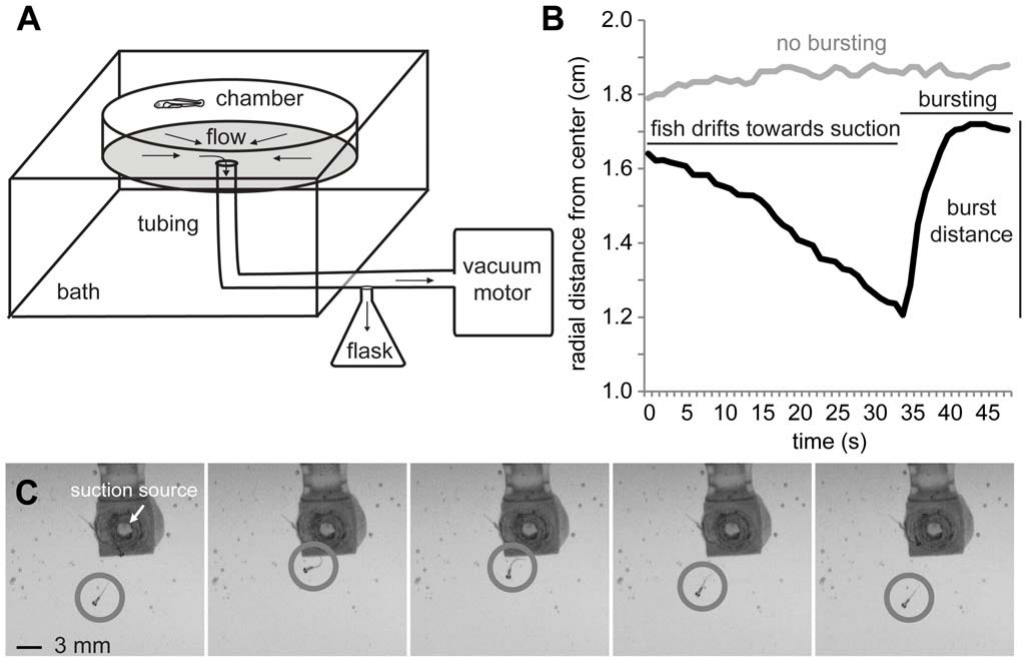
Larvae burst away from a continuous suction source at 5 days post fertilization. A. Schematic of the experimental suction chamber which is immersed in a large volume bath and connected with tubing to a retention flask and vacuum motor. B. Comparison of two suction avoidance behaviors. Position of a larva (black line) drifting towards and then bursting away from the suction source (origin at 0 cm), and another larva continuously holding station instead of bursting (gray line). C. Time series of a rheotactic larva (gray circles) escaping the suction source (arrow) with a quick burst of swimming. Frames taken every 350 ms.

Several species of predatory fishes and invertebrates prey on zebrafish using suction feeding, making the ability to detect and avoid high velocity flows critical for their survival [9]. Zebrafish are found in moderate flowing streams throughout Southeast Asia but congregate in slow flowing or stagnant side pools [9], [10]. The innate tendency to orient and swim against a current is called rheotaxis [11]. This behavior is mediated by the neuromasts of the lateral line system, and can proceed even in the absence of visual cues [11]. Although rheotaxis in adult fishes has received much attention [12]–[15], very little is known about rheotaxis and related behaviors in larvae. Here we employ a behavioral assay consisting of a controlled, continuous suction generator (Fig. 1A) in order to elicit rheotaxis and swimming bursts in 5 dpf larvae. We selectively ablated neuromasts to test the hypotheses that in the presence of a continuous flow, 1) the posterior lateral line is required to avoid a suction source, and 2) the span of neuromasts along the body is more important than the absolute number when avoiding a suction source. We describe a novel, lateral line mediated burst response and discuss how it may be part of a feedback circuit that relates burst distance to detected flow strength.

## Materials and Methods

### Animal Husbandry

Wildtype zebrafish (*Danio rerio*) were kept in a flow-through system (Aquatic Habitats, Apopka, FL, USA) at 28°C on a 14h∶10h light dark cycle. Similar conditions were maintained for larvae, which were kept in Petri dishes in an incubator (Tritech Research, Los Angeles, CA USA). All experiments were performed on 5 dpf zebrafish larvae in strict accordance with the recommendations in the Guide for the Care and Use of Laboratory Animals of the National Institutes of Health. The protocol was approved by the Institutional Animal Care and Use Committee of the University of Florida (Permit Number: 200903267 to J.C.L.).

### Experimental Suction Chamber

We constructed a suction chamber consisting of an 8.3 cm diameter, 7.7 cm tall cylindrical tank with an opening in the center of a mesh bottom, to which a 5 mm diameter nozzle (modified Luer Lock) was fitted. A section of 6 mm diameter Tygon tubing (Saint-Gobain Co., Akron OH USA) connected the nozzle to a 2000 mL Pyrex filter flask, and from there to a model 400–3910, 115 V, 60 Hz vacuum pressure motor (Barnant Company, Barrington, IL, USA). The vacuum motor was adjusted to remove 1.66±0.01 mL s^−1^ of fluid from the experimental chamber, so once turned on, a moderate suction flow was created at the location of the nozzle. Larvae which could not avoid the nozzle suction were captured and deposited into the filter flask.

The suction chamber was submerged in a larger, 3.3 L plastic container filled with Hank's solution in order to reduce the rate at which the water level dropped, while still maintaining a continuous suction. We empirically determined a flow velocity in which untreated larvae could routinely escape the suction source, which we then maintained with a 9 mm Hoffman clamp around the tubing.

### Selective ablation of neuromasts

Zebrafish larvae were anesthetized with tricaine sulfonate (Finquel, Redmont, WA) and exposed to 0.5 mM 2-(4-dimethylaminostyryl)-N-ethylpyridinium iodide (Sigma-Aldrich, St. Louis, MO, USA) in Hank's solution for 40 minutes in order to visualize the neuromasts. We developed a method to selectively ablate neuromasts by embedding an anesthetized fish in low melting point agar (Fisher Scientific, Fair Lawn, NJ, USA) and then carefully dissecting the solidified agar around targeted neuromasts. The exposed region was then bathed in 250 µM neomycin sulfate (Fisher Scientific, Fair Lawn, NJ, USA) for one hour. With this method the diffusion of neomycin through the agar could be minimized but not completely blocked; therefore our ablations involved subsets of neuromasts instead of individual neuromasts. We compared five experimental groups with different treatments: (1) anterior neuromasts of the posterior lateral line (PLL) ablated, (2) middle neuromasts of the PLL ablated, (3) caudal neuromasts of the PLL ablated, (4) complete PLL ablated and (5) a control group. The control group was embedded in agar and bathed in 10% Hank's solution for one hour instead of neomycin. Neuromast ablation was confirmed using an Olympus MVX10 fluorescent microscope (Center Valley, Pennsylvania, USA) under a 10× objective. After all treatments and sham procedures, larvae were placed in 10% Hank's solution for one hour to recover before testing in the suction chamber.

### Behavioral test

At the beginning of each experiment, a single larva was introduced into the chamber 1.5 cm away from the suction source. Larvae were scored as to whether they escaped or were captured by the suction source within a 10 second time frame. Behavior of individual larvae were recorded using a high-speed video camera (60 frames per second, 1200×800 pixel resolution, Phantom v12, Vision Research Inc., Wayne, NJ, USA). Initial experiments took place in a dark room with the suction chamber illuminated by a panel of infrared LEDs (BG Micro Garland, Texas). However, we discovered that performing the experiment in the light with a homogenous visual background produced the same results, so the remaining experiments were performed in the light.

Attempts to measure the radial flow velocity profile of the chamber by tracking small particles proved challenging with our setup. Therefore, the flow profile was determined by averaging the velocity of ten euthanized larvae drifting towards the suction source and smoothing the resulting curve with a cubic spline.

The ability for larvae to orient upstream in response to a current was analyzed by measuring the angle of the long body axis with respect to the flow direction for actively swimming fish compared to euthanized, passively drifting fish. Measurements started when the body axis was parallel with the flow direction (i.e. body angle = 0°). We then stepped back in ten-frame increments and measured the deviation between the body angle and this reference angle for each step. This yielded six body angle measurements that tracked the fish as it was orienting to the current over the course of one second.

### Data analysis and statistics

Videos were processed using a custom written MatLab program to track the position of the body over time (v12.1, Mathworks, Natick MA). Differences in the change in body axis angle were determined using an unpaired, one-tailed Student's T-test. Linear regression analyses revealed correlations between behavior and distance from the suction source, and their significance was tested at p<0.05. In the ablation experiments we used a Fisher's exact test to look for differences in the number of escaped and captured larvae between treatment groups.

## Results

We first characterized the burst response of 98 untreated larvae in the suction chamber The most common response was a single avoidance burst initiated when larvae approached the suction source. During the burst, a larva would move at least one third of its body length away from its starting location (Fig. 1B, 1C). Another, less common response was to maintain position with small, iterative movements to resist being drawn towards the suction source (Fig. 1B).

**Figure 2:**
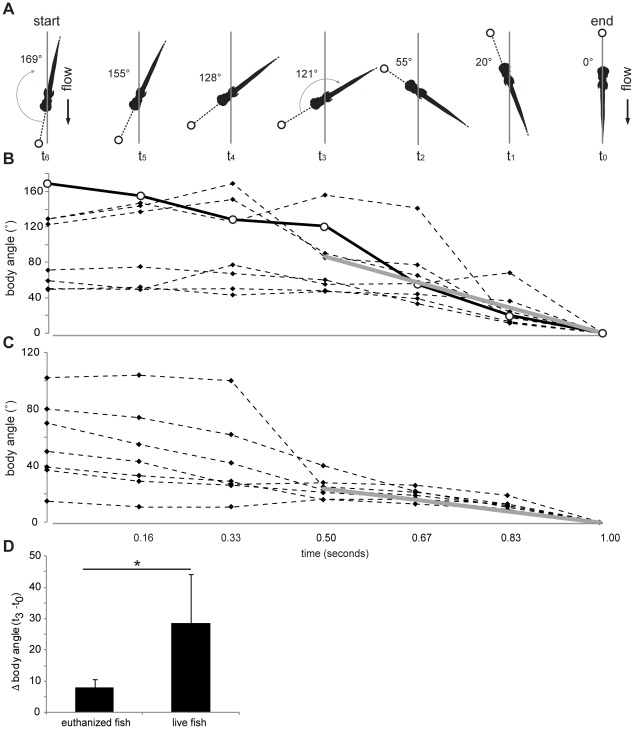
Larval zebrafish demonstrate rheotaxis by moving their body to orient upstream in the presence of current. A. Characteristic example of a larva turning its body upstream to align with a current created by a continuous suction source. The initial body angle relative to the flow direction (t_6_) decreases (gray arrows) with time such that at the end of a one second video sequence the body is aligned parallel to, and facing away from, the flow (t_0_). B. All body angles decreased over time in the presence of a current, indicating a robust rheotactic response in freely swimming larvae with an intact lateral line (n = 8 larvae). The data for larva in A are highlighted by white circles and joined by a solid black line. The solid gray line for the last three data points (highlighted in the black box) represents the average change in body axis between t_3_ and t_0_. C. Euthanized, and therefore passively drifting, larvae show a tendency to slowly self-orient to the current (n = 7 larvae). The solid gray line represents the average rate of change in body angle between t_3_ and t_0_. D. Comparison of the change in body angle between timepoints t_3_ and t^0^, when fish responded robustly to the flow. Live fish turn faster to align themselves with the flow than what is expected for a passively drifting fish (Student's unpaired, one-tailed T-test, * p<0.01, 7≤N≤8).

When fish initiated a burst, they actively oriented away from the suction source. At the end of a burst the body axis was aligned with the flow direction (Fig. 2A, 2B), thereby demonstrating a robust rheotaxis response. We also found that euthanized fish will passively align with the current generated by the suction source (Fig. 2C). However, the change in body angle is significantly faster in live, turning fish than in passively aligning fish (Fig. 2D).

**Figure 3:**
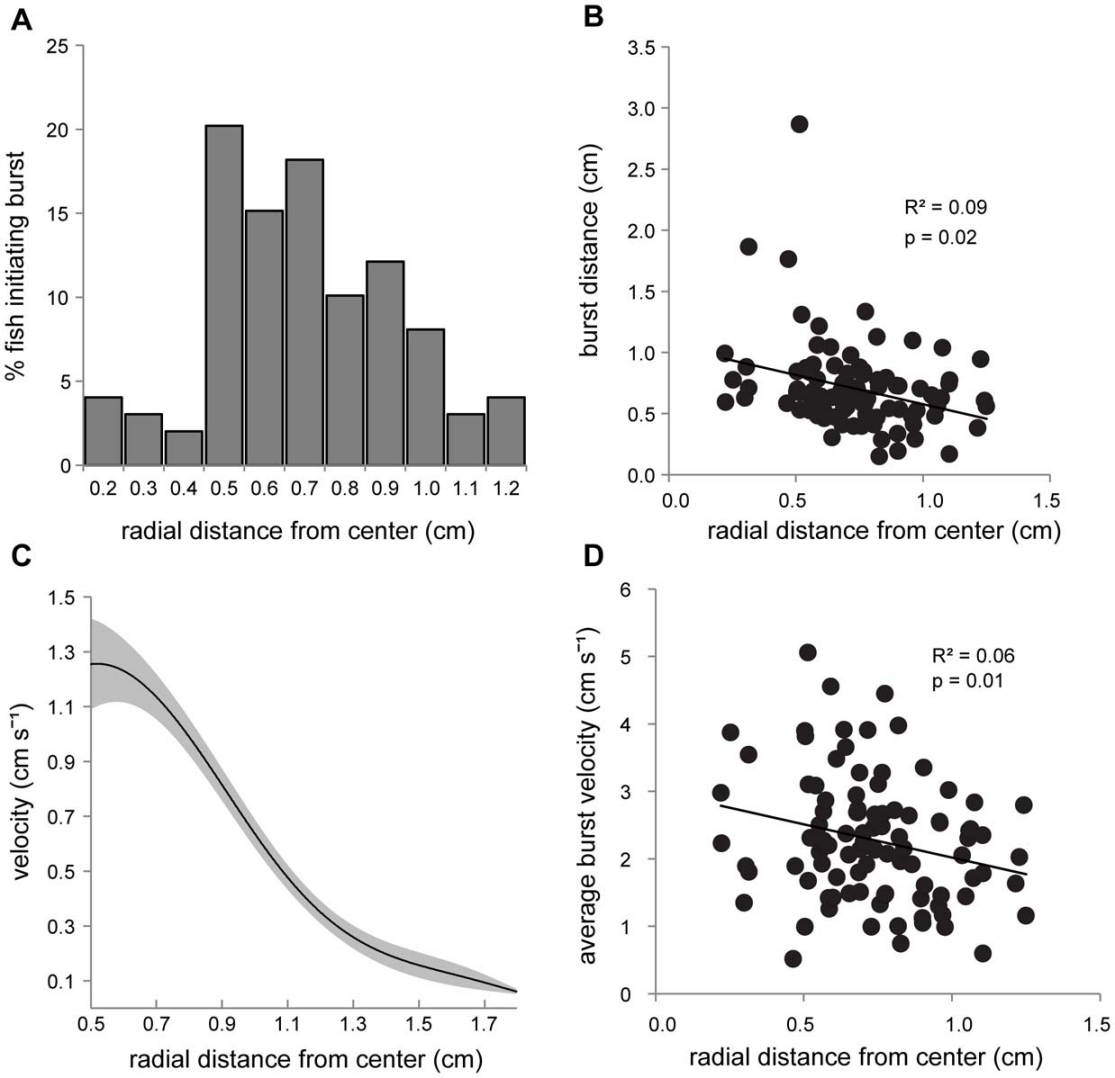
Characteristics of bursting behavior in the suction chamber for untreated larvae. A. Percentage of fish initiating a burst at a given radial distance from the suction source, where the origin of the suction source is at 0 cm. Most larvae initiate a swimming burst between 0.5–1.0 cm (1–2 body lengths) away from the suction source, while far fewer larvae initiate a burst closer or further away (N = 98). B. Relationship between burst distance and location of burst initiation. The closer to the suction source a burst is initiated the farther the distance traveled during the burst (N = 98, R^2^ = 0.09, p = 0.02). C. Velocity of passively drifting euthanized larvae as a function of distance from the suction source. The average drifting velocity of ten bodies was smoothed using a cubic spine (gray shaded area represents the standard error of the mean, N = 10). D. Average burst velocity as a function of the location of burst initiation. The closer to the suction source the burst is initiated, the faster the burst velocity (N = 98, R^2^ = 0.06, p = 0.01).

To determine the distance from the suction source at which larvae initiated an avoidance burst, we marked the chamber into one millimeter radial sections from the center. Figure 3A shows a frequency distribution of the distances from the suction source at which larvae initiated a burst. Most larvae initiated a burst between 0.5 and 1.0 cm from the suction source. Far fewer larvae initiated a burst when located closer to the suction source, or when they were more than 1 cm away from the suction source. Larvae that initiated a burst closer to the suction source tended to travel a greater distance during the burst than larvae that initiated their burst further away from the suction. Therefore, the location of burst initiation and the burst distance traveled is significantly correlated (Fig. 3B). We assume that larvae located closer to the suction source experienced faster flows than larvae located further away from the suction source, based on the velocity values that we measured with euthanized larvae (Fig. 3C). Larvae that initiated their burst closer to the suction source had a higher burst velocity compared to larvae that initiated a burst further away. The result is that the average burst velocity was also significantly correlated to the location of the burst initiation (Fig. 3D).

**Figure 4:**
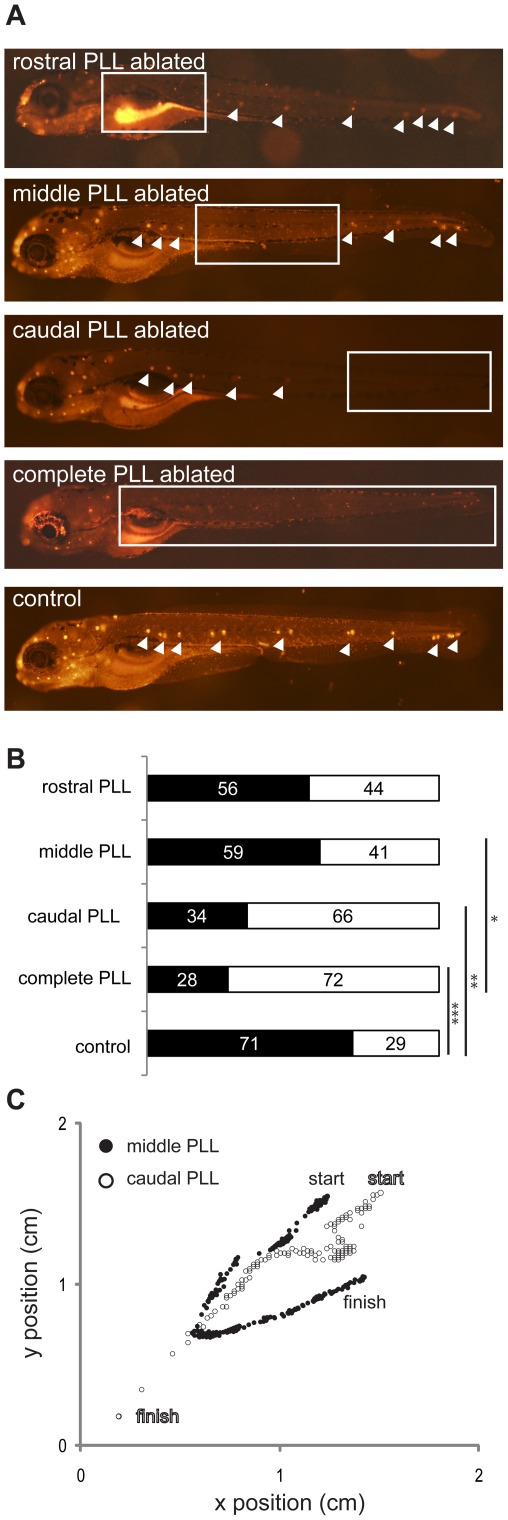
The effects of selective neuromast ablation on the ability of larvae to avoid a suction source. A. DASPEI-labeled neuromasts in 5 day post fertilization larvae with sections of the posterior lateral line (PLL) ablated with neomycin. Intact neuromasts labeled with DASPEI are highlighted with white arrowheads, while white boxes indicate regions where neomycin was applied. Note that due to the transparency of the larvae, at times labeled neuromasts from the opposite side of the body are seen. Five different treatments were tested, from top to bottom: (1) larvae with rostral neuromasts of the PLL ablated, (2) middle neuromasts of the PLL ablated, (3) caudal neuromasts of the PLL ablated, (4) complete PLL ablated and (5) sham treated control group. B. Percent larvae that escape (black bars) and are captured by (white bars) the suction source. There is a significant difference between the control (N = 78) and the complete PLL ablated group (N = 36) as well as between the control and the caudal neuromasts ablated group (N = 32). We also found a significant difference between the complete PLL ablated group and middle neuromasts ablated group (N = 27). No significant effects where found for the rostral neuromasts ablated group (N = 18). All groups were tested using a Fisher's exact test (***p<0.001, **p<0.01, *p<0.05). C. Time series showing the position of a larva with caudal neuromasts ablated (white circles) captured by the suction source (located at the origin of the coordinate system), and a larva with middle neuromasts ablated (black circles) bursting away from the suction source. Start and endpoint of each path are indicated.

We next asked which neuromasts of the posterior lateral line system (PLL) are involved in detecting the flow created at the suction source. Subsets of PLL neuromasts were ablated with neomycin to create five treatment groups, comprised of fish with (1) rostral neuromasts of the PLL ablated, (2) middle neuromasts of the PLL ablated, (3) caudal neuromasts (neuromasts posterior to and including the L5) of the PLL ablated, (4) the complete PLL ablated and (5) a control group which went through a sham ablation procedure but had an intact PLL (Fig. 4A). In all groups the anterior lateral line system containing the cranial neuromasts was left intact. Treatments 1–3 were designed to affect approximately the same number of neuromasts. We found that significantly fewer larvae escaped the suction source when either the complete PLL was ablated or the caudal neuromasts were ablated compared to control larvae (Fig. 4B). There was no significant difference between control larvae and larvae with either their rostral or middle PLL neuromasts ablated. We found a significant difference between larvae with their middle neuromasts ablated and larvae with their complete PLL ablated. There were no significant differences between complete PLL ablated larvae and larvae with only their caudal neuromasts ablated.

The swimming trajectories of larvae under different treatments differed considerably. A larva with its caudal neuromasts ablated typically drifts towards and is eventually captured by the suction source, while a larva with its middle neuromasts ablated can initiate an avoidance burst to avoid the suction source (Fig. 4C).

## Discussion

Rheotaxis in adult fishes is defined as the ability to orient upstream to current and relies on the neuromasts of the lateral line system [16]. Larval zebrafish display many well-characterized locomotor behaviors [4], but surprisingly few studies examine how larvae behave in moving water [7] despite the fact that in their natural habitat they are found in streams with flows documented at 0.07±0.05 m s^−1^ [10]. The ability for a flow stimulus to initiate motor behavior is not unprecedented. In still water, a sufficiently strong hydrodynamic stimulus from the lateral line activates the paired Mauthner cells in the hindbrain to generate a robust, all-or-nothing escape response in the absence of input from other modalities [6], [7]. We demonstrate that larval zebrafish are rheotactic by showing that they actively orient towards the flow direction faster than the passive aligning of euthanized fish (Fig. 2).What makes our results remarkable are that we demonstrate for the first time that 1) superficial neuromasts of the posterior lateral line system are used for rheotaxis in fish larvae, similar to what has been found for *Xenopus* larvae [17], and 2) even during rheotaxis superficial neuromasts play a fundamental role in the ability to detect and burst away from accelerating flows created by a suction source.

Larvae that initiated a burst closer to the suction source, where flow is faster, swam faster and farther than larvae that initiated a burst further away from the suction source, where flow is slower (Fig. 3B and 3D). We can imagine at least two scenarios to explain this behavior. One possibility is that the distance that larvae burst is a preprogrammed motor response which is positively correlated to the magnitude of the flow velocity sensed prior to the burst. This scenario assumes no feedback during the burst; the stronger the initial flow, the further the burst distance traveled (Fig. 3B). This applies to larvae that start their burst closer to the suction source. The result is that regardless of whether larvae initiate their bursts closer to, or further away from, the suction source, both can end up at a similar distance away. Another possibility is that larvae can continuously detect flow while bursting, and only stop when they sense low flow. Assuming a homogenous radial flow profile in the suction chamber, all larvae would then terminate their burst at a similar distance away from the suction source regardless of where they initiate the burst. The limitations of our experiment make it difficult to evaluate if one mechanism is involved or a combination of the two. This question could be addressed in a future study by locally introducing higher flow to larvae located farther away from the suction source (where flow would be relatively low). If larvae end their bursts in the same region of the chamber as larvae located closer to the suction source, then this would support the interpretation that larvae only stop when they sense low flow.

Our behavioral results lead us to predict that the spatial array of posterior lateral line neuromasts is functionally integrated with the topography of spinal cord neurons to take part in initiating and modulating a range of swimming speeds. Previous work demonstrates that dorsal, glutamatergic neuropil regions in the hindbrain are associated with fast swimming networks in the spinal cord, compared to more ventral neuropil regions [18], and that caudal neuromasts connect more dorsally in the hindbrain than rostral neuromasts [19]. According to this hypothesis, during rheotaxis stimulated neuromasts would inform a class of excitatory spinal interneurons that are responsible for slow swimming (i.e. multipolar, commissural descending interneurons, MCoDs). Higher velocity flows, such as those found closer to the suction source, would stimulate neuromasts that are connected to fast-swimming networks involving another class of glutamatergic, circumferential descending interneurons (CiDs).

By selectively ablating certain neuromasts along the body trunk while leaving others intact, we discovered that not all neuromasts are functionally equal (Fig. 4). This makes it challenging to evaluate our original hypothesis; that neuromast span is more important than absolute number in order to avoid a suction source. Instead, our results lead us to conclude that caudal neuromasts seem to be disproportionately responsible for initiating the burst response, such that even the removal of the entire PLL does not change larval performance significantly beyond the removal of caudal neuromasts (Fig. 4B). Caudal neuromasts have a unique development and morphology that reveals important functional implications. For example, the afferent neurons that innervate caudal neuromasts are known to have larger and more complex growth cones than neurons that innervate more anterior neuromasts [19], and are the first to differentiate [20].

Caudal neuromasts likely play a key role in detecting flow velocity and direction in order to orchestrate the burst response in larvae. Interestingly, rostral neuromasts of the posterior lateral line system may play a more prominent role in escape bursts than middle neuromasts, given that a significant difference exists between middle and complete ablation treatments but not between rostral and complete ablation treatments. More systematic ablation and recording experiments will advance our understanding of how flow information is processed. We envision that flow detection could also proceed by a mechanism that calculates the difference in rostral and caudal neuromast stimulation times. This has been modeled, but found challenging to attack empirically, in adult fish due to the enormous numbers of neuromasts present [21]. Detection of flow could also operate under models applied to directional sensing in electric fishes [22]. We believe that the simple and accessible lateral line system in larval zebrafish provides a strong approach to better understanding how flow is translated into motor behaviors at the single cell level.
